# Methemoglobinemia With Dapsone Prophylaxis in a Patient With Minimal Change Disease

**DOI:** 10.7759/cureus.26393

**Published:** 2022-06-28

**Authors:** Manuel J Rovira Gonzalez, Robert Pargament

**Affiliations:** 1 Internal Medicine, WellSpan York Hospital, York, USA

**Keywords:** methemoglobinemia, pneumocystis jiroveci pneumonia prophylaxis, minimal change disease, nephrotic syndrome, methylene blue, dapsone, hypoxemia, dyspnea, shortness of breath

## Abstract

Our case report represents the need to maintain vigilance for methemoglobinemia risk in patients without classic symptoms, specifically, in patients that develop shortness of breath after starting prophylaxis for *Pneumocystis jiroveci* pneumonia, mostly with dapsone.

A case report of a 42-year-old male with minimal change disease nephrotic syndrome required *Pneumocystis jiroveci* pneumonia prophylaxis due to high-dose systemic steroids. The patient was started on dapsone due to side effects and the availability of alternative medications. Since starting therapy, the patient developed progressive dyspnea upon exertion for two weeks with intermittent hypoxia. The patient tested negative for glucose-6-phosphate dehydrogenase deficiency prior to starting dapsone. He was also on therapeutic enoxaparin due to a hypercoagulability state from nephrotic syndrome. The patient presented with hypoxia and dyspnea upon exertion, however, speaking in complete sentences and with no cyanosis or overt findings of hypervolemia. The patient remained hypoxemic despite supplemental oxygen. An arterial blood gas was performed and showed methemoglobin levels of 10.6 percent. He was treated with methylene blue with the resolution of methemoglobinemia and hypoxemia after a second dose. Trimethoprim-sulfamethoxazole was started for *Pneumocystis jiroveci* pneumonia prophylaxis. He was safely discharged home.

## Introduction

Methemoglobinemia is a blood disorder that can be acquired or congenital, acquired form being the most common [1]. It has been most associated with medication and anesthetic use, dapsone and benzocaine being the most common agents [1-3]. Methemoglobinemia leads to tissue hypoxia by creating ferric (Fe3+) hemes where oxygen is unable to bind, increasing the affinity of oxygen to hemoglobin, and therefore, reducing oxygen delivery to tissue. Patients can present with subtle findings such as cyanosis and dark brown-colored arterial blood [1]. Left untreated, it can potentially lead to coma and lethal arrhythmia [4].

Methemoglobinemia develops because of the inability of methemoglobin to be reduced at an equal or greater rate than is being produced. This can happen by increased oxidation of hemoglobin from drugs or oxidative stress or by a deficiency in reducing capacity such as cytochrome b5 reductase deficiency or decreased nicotinamide adenine dinucleotide phosphate (NADPH) production. Dapsone is primarily used for prophylaxis against *Pneumocystis jiroveci* pneumonia but also is used in dermatology and inflammatory bowel disease [1]. Dapsone’s potent hydroxylated amine oxidant metabolites cause its adverse effects including methemoglobinemia and hemolytic anemia [4]. Clinical presentation of methemoglobinemia results in refractory hypoxemia, cyanosis, and dark-colored arterial blood [1].

## Case presentation

A 42-year-old male presented to the emergency room with progressive dyspnea for two weeks. His past medical history was significant for recently relapsed minimal change disease, hypothyroidism due to Hashimoto’s thyroiditis, and Gilbert’s syndrome. During a recent hospitalization, the patient was started on high-dose prednisone after a kidney biopsy showed minimal change disease. Dapsone 100 mg daily was added for *Pneumocystis jiroveci* pneumonia prophylaxis. He was not started on trimethoprim-sulfamethoxazole because of acute kidney injury. Atovaquone was not used due to a national shortage. The patient was also started on prophylactic enoxaparin due to an increased clotting burden from nephrotic syndrome. The patient returned to the emergency room two weeks after being discharged with dyspnea upon exertion and having desaturation at home of 83 to 87 percent.

He did not have weight gain, orthopnea, bendopnea, calf swelling or tenderness, dizziness, fever, palpitations, chest pain, or cough. The remainder of the review of systems was negative.

His home medications included prednisone, levothyroxine, prophylactic enoxaparin, bumetanide, and dapsone.

Upon examination, he was well-appearing, speaking in complete sentences and in no acute distress. He was afebrile, normal respiratory rate of 14 breaths per minute, and normotensive, however, with intermittent hypoxia at 87 percent while on four liters of oxygen through a nasal cannula. The patient had a regular cardiac rhythm, no murmurs, clear breath sounds bilaterally, no jugular vein distention, no calf swelling or tenderness, no cyanosis, and capillary refill less than two seconds.

Laboratory results showed mild leukocytosis of 11.3 K/mcL, hemoglobin 13.1 g/dL, and platelets 208 K/mcL. Creatinine was 1.12 mg/dL, at baseline. Urinalysis was within normal limits.

Arterial blood gas showed pH 7.52, partial CO2 34 mmHg, partial O2 90 mmHg, oxyhemoglobin saturation of 87 percent, and methemoglobin levels of 10.6 percent. A pulse saturation of 100 percent at the moment of arterial blood gas with an arterial oxygen saturation of 87 percent indicated the presence of an oxygen saturation gap, the difference between pulse and arterial saturation, of 13 percent.

C-reactive protein and B-type natriuretic peptide were less than 1 mg/L and 32 pg/mL, respectively. Urine toxicology screen was negative, thyroid-stimulating hormone levels were 22.38 mcIU/mL, and free T4 levels of 1.2 ng/dL. HIV and COVID-19 PCR were negative. Lactate dehydrogenase was within limits.

The patient’s chest X-ray and electrocardiogram were within normal limits. A recent echocardiogram was normal.

The patient was given an injectable 1 percent solution of methylene blue (1 mL/kg) twice, with a resolution of methemoglobinemia and oxygen requirements after the second dose. The patient was monitored overnight, and trimethoprim-sulfamethoxazole was started for prophylaxis in lieu of dapsone.

## Discussion

Methemoglobin is an abnormal hemoglobin that contains oxidized iron (ferric, or Fe3+) rather than normal iron (ferrous, or Fe2+). Ferric-based heme is unable to bind oxygen and also serves to cause a left shift in the hemoglobin dissociation curve. Both of these factors contribute to decreased oxygen delivery to tissues. Methemoglobinemia is diagnosed at levels greater than 3 percent; tissue hypoxia typically develops at levels greater than this. Levels greater than 15 percent are associated with cyanosis [4]. Fatal arrhythmias can develop in levels greater than 70 percent [5]. Tissue hypoxia usually develops at methemoglobin levels greater than 3 percent [5].

Acquired methemoglobinemia due to medications is the most common cause of methemoglobinemia; dapsone is the most common agent as found by a retrospective study [2]. Other medications associated with acquired methemoglobinemia include sulfonamides, chloroquine, benzocaine, lidocaine, nitroprusside, rasburicase, and rifampin [1,2,6]. Dapsone is a sulfone antibiotic with anti-inflammatory properties mostly used for *Pneumocystis jiroveci* pneumonia prophylaxis. It is also used to treat dermatitis herpetiformis, leprosy, toxoplasmosis, ulcerative colitis, as well as immune thrombocytopenic purpura [4,6]. Additionally, patients with anemia are at higher risk of developing methemoglobinemia [6] while blood transfusions or exchange can decrease levels [5].

Treatment for methemoglobinemia involves removing the offending agent and administering methylene blue to symptomatic patients or those with methemoglobin levels greater than 30 percent [1,4]. Methylene blue acts to reduce methemoglobin (Fe3+) to hemoglobin (Fe2+) via NADPH. Ascorbic acid should be started on patients with suspicion of glucose-6-phosphate dehydrogenase deficiency or if methylene blue is otherwise contraindicated or unavailable [1]. Patients usually respond to treatment with methylene blue within 60 minutes; treatment can be repeated if needed [1,6]. In our case, an additional dose of methylene blue was given to the patient for persistent symptoms thought to be related to the extended half-life of dapsone metabolites (i.e., hydroxylated amine metabolites), which are potent oxidants that cause dapsone’s adverse effects including methemoglobinemia and hemolytic anemia [4].

If dapsone is not tolerated, patients can be transitioned to either atovaquone or trimethoprim-sulfamethoxazole for *Pneumocystis jiroveci* pneumonia prophylaxis. Trimethoprim-sulfamethoxazole is the most cost-effective prophylaxis; atovaquone is a more expensive choice but can be used if there are contraindications to either of the other two options [7].

It is unclear if nephrotic syndrome increases the risk of methemoglobinemia due to dapsone. Two additional cases of dapsone-induced methemoglobinemia in patients with nephrotic syndrome have been recently reported [6,8]. We want to raise awareness of this possible association so that patients can be monitored closely for this side effect. Further research is needed to understand the pathophysiology in dapsone-induced methemoglobinemia in patients with nephrotic syndrome.

## Conclusions

Nephrotic syndrome patients can present with methemoglobinemia without classical symptoms. However, desaturations, a saturation gap, and shortness of breath are other signs and symptoms to be aware of in the absence of cyanosis. We reiterate the need to consider methemoglobinemia when a nephrotic syndrome patient presents with shortness of breath within one month of starting dapsone even in patients without glucose-6-phosphate dehydrogenase deficiency. Trimethoprim-sulfamethoxazole and atovaquone are alternatives if patients are intolerant to dapsone.
